# Buckwheat-Associated Acute Psychosis: A Case of Anticholinergic Syndrome

**DOI:** 10.7759/cureus.74641

**Published:** 2024-11-27

**Authors:** Daniela Barbosa Mateus, Carolina Henriques Gomes, João Barbosa Barroso, Sofia Cruz

**Affiliations:** 1 Internal Medicine, Hospital Vila Franca de Xira, Vila Franca de Xira, PRT; 2 Medicine, Hospital Vila Franca de Xira, Vila Franca de Xira, PRT

**Keywords:** acute psychosis, anticholinergic syndrome, buckwheat, neuropsychiatric disorders, tropane alkaloids

## Abstract

Buckwheat (*Fagopyrum esculentum*) is a seed increasingly used as a gluten-free alternative, particularly by individuals with gluten-sensitive enteropathy. While rich in vitamins and minerals, it may also contain toxic secondary metabolites.

The authors present a case of a 49-year-old female patient, admitted to the emergency department with a four-hour history of psychomotor agitation, confusion, and mydriasis. Symptom onset occurred three hours after consuming buckwheat. Clinical examination revealed disorientation, agitation, and visual and auditory hallucinations, with mydriasis and tachycardia. Laboratory and imaging tests were unremarkable. Toxicology screening was negative for common substances of abuse. A diagnosis of acute psychosis secondary to buckwheat ingestion was made. Management included intravenous diazepam, leading to a favorable clinical outcome.

Buckwheat may contain tropane alkaloids, which can be toxic to humans. Benzodiazepines were effective in managing acute psychosis. This case highlights the potential neuropsychiatric effects of buckwheat ingestion and the importance of considering dietary causes in the differential diagnosis of acute psychosis.

## Introduction

Buckwheat (*Fagopyrum esculentum*) is a pseudocereal widely consumed as a gluten-free alternative, especially among individuals with gluten-sensitive enteropathy. It is valued for its nutritional profile and is rich in essential vitamins, minerals, and bioactive compounds [1,2].

Buckwheat can be contaminated by *Datura *spp, which can contain high concentrations of tropane alkaloids [3]. Certain tropane alkaloids possess pharmacological properties and may function as anticholinergics or stimulants [4].

Although generally considered safe, there is limited literature on its potential to induce neuropsychiatric symptoms, particularly psychosis characterized by hallucinations and delirium.

This report presents a rare case of acute psychosis following buckwheat ingestion, emphasizing the need for awareness of the potential toxicological effects associated with this increasingly popular food.

## Case presentation

A 49-year-old female patient with a medical history of hypothyroidism managed with 100 mcg of levothyroxine daily, presented to the Emergency Department with a four-hour history of psychomotor agitation, mental confusion, and mydriasis. The patient reported consuming pancakes made with buckwheat approximately three hours before symptom onset. There was no prior history of allergies.

On physical examination, the patient appeared disoriented and agitated, exhibiting visual and auditory hallucinations. Her pupils were bilaterally dilated and reactive to light. She was normotensive, tachycardic (heart rate: 140 beats per minute), afebrile, and without signs of respiratory distress. The Glasgow Coma Scale (GCS) score was 15, indicating full consciousness, with no focal neurological deficits observed.

**Figure 1:**
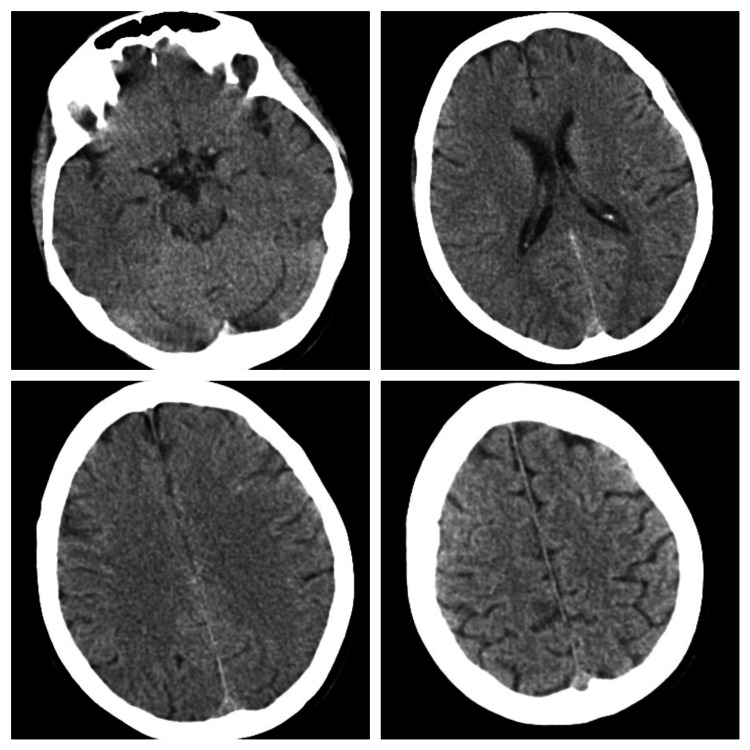
Cranial CT scan

Initial laboratory investigations (Table 1) were unremarkable, including a metabolic panel and thyroid function tests. Urine toxicology screening was negative for amphetamines, benzodiazepines, cannabinoids, cocaine derivatives, opiates, and methamphetamines. A cranial CT scan was performed without changes (Figure 1).

**Table 1:** Laboratory findings on the day of admission to the emergency room pCO2: partial pressure of carbon dioxide, pO2: partial pressure of oxygen, HCO3: bicarbonate

Test	Value	Reference range	Comment
Hemoglobin	13,2 g/dL	12-15 g/dL	Normal
White blood cells	6300/µL	4000-1000/µL	Normal
Platelets	248000/µL	150000-400000/µL	Normal
C-reactive protein	<0,5 mg/dL	< 1 mg/dL	Normal
Creatinine	0,70 mg/dL	0.7-1.3 mg/dL	Normal
Urea	32 mg/dL	< 50 mg/dL	Normal
Sodium	137 mmol/L	136-145 mmol/L	Normal
Potassium	4.24 mmol/L	3.5-5.1 mmol/L	Normal
Total bilirubin	0.35 mg/dL	0.1-1.2 mg/dL	Normal
Alanine transaminase	35 U/L	< 59 U/L	Normal
Aspartate transaminase	30 U/L	< 50 U/L	Normal
Thyroid-stimulating hormone	2.886 mU/L	0.350-5.5 mU/L	Normal
Free T4	1.42 ng/dL	0.8-1.76 ng/dL	Normal
Creatine phosphokinase	93U/L	26-192 U/L	Normal
Urinary test for drugs	Negative to amphetamines, benzodiazepines, cannabinoids, cocaine derivatives, opiates, and methamphetamines		
Arterial blood gases			
pH	7.47	7.35-7.45	Above normal
pCO2	36.2 mmHg	35-45 mmHg	Normal
pO2	108.7 mmHg	> 65 mmHg	Normal
HCO3	25.8 mmHg	22-26 mmHg	Normal
Lactate	2.01 mmol/L	< 1.8 mmol/L	Above normal

Given the temporal association with buckwheat consumption and the absence of other identifiable causes, a diagnosis of acute psychosis induced by buckwheat toxins was made. The Poison Information Center was consulted, and benzodiazepine administration was recommended, as physostigmine was unavailable. The patient received a total of 9 mg of intravenous diazepam. 

She was closely monitored in the emergency department, and her symptoms gradually improved. During this period, the patient was re-evaluated, showing continued resolution of her agitation and hallucinations. After 12 hours, she was stable, alert, and oriented, with no further neuropsychiatric symptoms.

## Discussion

In recent years, there has been a rise in the inclusion of functional foods with bioactive compounds in consumers' diets. These foods provide both nutritional value and health benefits to consumers [1]. 

Buckwheat is known to contain various bioactive compounds, including flavonoids and phytosterols, and may have secondary metabolites such as tropane alkaloids, which can be toxic [1]. Plants are also a common source of both accidental and intentional anticholinergic ingestion. Plants containing belladonna alkaloids, such as scopolamine and, more prominently, atropine, may exhibit hallucinogenic properties due to their antimuscarinic effects [5]. These compounds can cause a range of symptoms, including delirium, confusion, tachycardia, and dilated pupils, and are often misused for their psychoactive effects [6,7]. Buckwheat can be contaminated by *Datura* spp., which contains high levels of tropane alkaloids, and can contaminate buckwheat crops if they grow nearby or if the seeds come into contact [3].

Similarly with this case, in 2003, Slovenia witnessed a case of mass poisoning affecting 73 individuals, who exhibited symptoms including dry mouth, flushed skin, blurred vision, tachycardia, urinary retention, ataxia, speech disturbances, disorientation, and visual hallucinations [7]. All individuals, who were affected, had ingested food products made with buckwheat flour in the hours leading up to the appearance of symptoms. A subsequent investigation revealed that the poisoning was caused by the inadvertent contamination of the buckwheat flour with *Datura stramonium* seeds, leading to anticholinergic toxicity [7].

Management of anticholinergic toxicity typically involves benzodiazepine administration intravenously to control mild to moderate agitation [8]. In cases of hypotension or suspected rhabdomyolysis, intravenous fluids should be administered. If significant hyperthermia is present, cooling interventions should be initiated. Additionally, activated charcoal may be considered if the ingestion occurred within one hour before the patient’s presentation [5]. In severe cases, cholinesterase inhibitors, such as physostigmine, may be considered. As a reversible acetylcholinesterase inhibitor, physostigmine increases acetylcholine concentration at the synaptic cleft, serving as an antidote for anticholinergic syndrome by effectively reversing toxicity in both the central and peripheral nervous systems [6].

In this case, despite the unavailability of physostigmine, diazepam effectively managed the acute psychotic symptoms, highlighting the importance of timely intervention. Usually, when anticholinergic toxicity is promptly recognized and proper supportive treatment is provided, the outcome is favorable [5].

This case underscores the importance of considering dietary and environmental factors in the differential diagnosis of acute psychosis, especially in patients without a prior psychiatric history. Although buckwheat is generally considered safe, healthcare providers should know its potential to induce toxic effects.

## Conclusions

This case illustrates a rare but significant neuropsychiatric complication following buckwheat ingestion, emphasizing the need for increased awareness of its potential toxic effects. While buckwheat is typically considered a safe and nutritious pseudocereal, the presence of tropane alkaloids may contribute to adverse reactions such as acute psychosis. Prompt recognition and treatment with benzodiazepines, as demonstrated in this case, can lead to a favorable outcome. As the consumption of functional foods like buckwheat increases, healthcare providers should remain vigilant for rare but serious toxicological effects.
